# Targeting MCL-1 in cancer: current status and perspectives

**DOI:** 10.1186/s13045-021-01079-1

**Published:** 2021-04-21

**Authors:** Haolan Wang, Ming Guo, Hudie Wei, Yongheng Chen

**Affiliations:** 1 Department of Oncology, NHC Key Laboratory of Cancer Proteomics, Laboratory of Structural Biology, National Clinical Research Center for Geriatric Disorders, Xiangya Hospital, Central South University, Changsha, 410008 Hunan China; 2 National Clinical Research Center for Geriatric Disorders, Xiangya Hospital, Central South University, Changsha, 410008 Hunan China

**Keywords:** MCL-1, BCL-2 family, Cancer, Apoptosis, Inhibitor

## Abstract

Myeloid leukemia 1 (MCL-1) is an antiapoptotic protein of the BCL-2 family that prevents apoptosis by binding to the pro-apoptotic BCL-2 proteins. Overexpression of MCL-1 is frequently observed in many tumor types and is closely associated with tumorigenesis, poor prognosis and drug resistance. The central role of MCL-1 in regulating the mitochondrial apoptotic pathway makes it an attractive target for cancer therapy. Significant progress has been made with regard to MCL-1 inhibitors, some of which have entered clinical trials. Here, we discuss the mechanism by which MCL-1 regulates cancer cell apoptosis and review the progress related to MCL-1 small molecule inhibitors and their role in cancer therapy.

## Introduction

The orderly and delicate regulation of apoptosis of cells under physiological and pathological conditions is an autonomous clearance mechanism adopted by cells to maintain their own homeostasis [1]. Under normal circumstances, cell growth, proliferation and death maintain a dynamic balance to ensure cellular homeostasis and normal physiological function. When an organism is subjected to certain stimuli that disrupt this balance, it may cause cell proliferation to outpace apoptosis, potentially even leading to tumorigenesis.

**Fig. 1:**
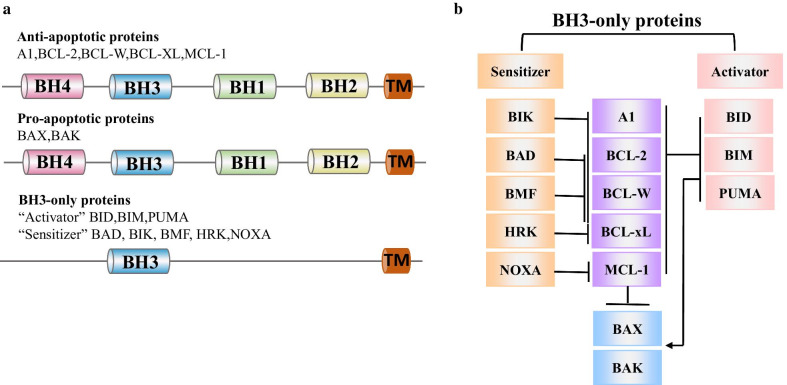
An overview of BCL-2 family proteins. **a** Schematic representation of the domains of BCL-2 family proteins. Anti-apoptotic proteins and pro-apoptotic effector proteins both contain BH domains 1–4 (BH 1–4). BH3-only proteins contain only the BH3 domain (except for BID, which has BH1-4). **b** The interactions of BCL-2 family proteins. Antiapoptotic proteins prevent the activation of the effector proteins BAX and BAK. BH3-only proteins are divided into “sensitizers” and “activators”. “Activators” directly activate BAX and BAK and inhibit antiapoptotic proteins. “Sensitizers” only suppress anti-apoptotic proteins. The specific interaction network is shown

There are two apoptotic pathways: the intrinsic (or mitochondrial) pathway of apoptosis and the extrinsic pathway of apoptosis. The mitochondrial pathway is activated by intracellular signals and is strictly controlled by the BCL-2 family [2]. BCL-2 family proteins share one or four BCL-2 homologous domains (BH 1–4) and are grouped into three subsets (Fig. 1a) [3, 4]: antiapoptotic BCL-2 proteins (BCL-2, BCL-xL, BCL-W, MCL-1 and BCL-2-related gene A1), proapoptotic effectors (BAX and BAK) and BH3-only proteins. The BH3-only proteins are subdivided into the “activator” (BIM, BID and PUMA) and the “sensitizer” (BAD, BIK, BMF, HRK and NOXA) [5]. These BCL-2 family proteins function through complex interactions to regulate the integrity of the mitochondrial membrane (Fig. 1b).

Myeloid leukemia 1 (MCL-1) is an antiapoptotic member of the BCL-2 family. MCL-1 inhibits mitochondrial outer membrane permeabilization (MOMP) and the release of cytochrome C from mitochondria. MCL-1 is necessary for the survival of many cells, such as the nervous system [6], T/B lymphocytes [7], and cardiomyocytes [8]. MCL-1 also has high oncogenic potential and is upregulated in a range of malignancies, including solid tumors and hematological malignancies [9]. Overexpression of the MCL-1 protein or amplification of the MCL-1 gene protects cancer cells from apoptosis and decreases their sensitivity to commonly used anticancer drugs, which has emerged as a resistance mechanism against multiple anticancer therapies, including radiotherapy, chemotherapy, and BH3 mimics targeting BCL-2/BCL-XL [10, 11]. Therefore, MCL-1 is a very promising target for tumor treatment.

In this review, we describe the known functions of MCL-1 in normal and malignant cells and discuss the development and clinical trials of some MCL-1 small molecule inhibitors in the quest for new anticancer drugs.

### Isoforms of MCL-1 protein

**Fig. 2:**
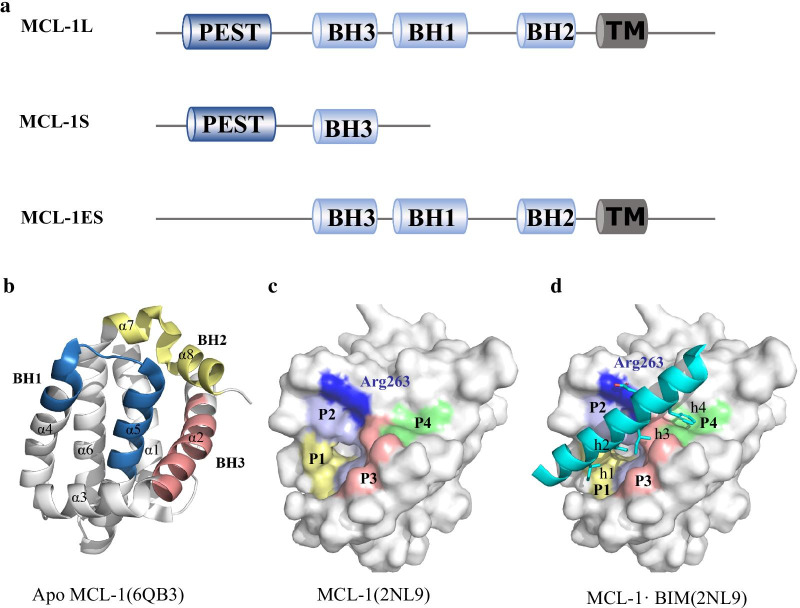
The isoforms and three-dimensional structure of MCL-1. **a** Three splicing variants of the human MCL-1 gene. **b** Cartoon representation of the tertiary structure of MCL-1 (PDB ID: 6QB3). The BH core of MCL-1 is composed of eight α helices. The BH1 (blue), BH2 (yellow) and BH3 (red) domains form a hydrophobic groove called the BH3-binding groove. **c** Surface representation of the BH3 binding groove of MCL-1 (PDB ID: 2NL9). The four hydrophobic pockets (P1–4) are highlighted and labelled. **d** Structure of the MCL-1/BIM complex (PDB ID: 2NL9). MCL-1 is shown as surface. BIM is shown as cyan cartoon. The four hydrophobic residues of BIM (h1, h2, h3, h4) are shown as cyan sticks

There are three splicing variants of the human MCL-1 gene, including MCL-1L, MCL-1S and MCL-1ES (Fig. 2a). The three splice variants play distinct roles in apoptosis. The long variant (MCL-1L) encoded by exons I to III of the MCL-1 gene acts as an anti-apoptotic factor, and MCL-1L is traditionally referred to as MCL-1. MCL-1 shares four BH domains. Similar to other multidomain BCL-2 proteins, the tertiary structure of the BH core of MCL-1 is composed of eight α helices (Fig. 2b). The BH1 domain constitutes turn regions linking helices 4 and 5, and the BH2 domains constitute turn regions linking helices 7 and 8. The BH3 domain is located in helix 2, and the BH4 domain is located in helix 1. Helices 2–5 and 8 constitute a hydrophobic groove (called the BH3-binding groove) that is critical for its interactions with the BH3 domain of proapoptotic BCL-2 members (Fig. 2c, d) [12]. MCL-1 also has a transmembrane α-helix domain at the C-terminus, allowing it to localize to the cell membrane, especially the outer mitochondrial membrane [13]. In particular, MCL-1 has a large N-terminal region with a PEST domain rich in proline (P), glutamic acid (E), serine (S), and threonine (T). Multiple sites of phosphorylation, ubiquitination, and caspase enzymatic cleavage are present within the PEST domain, allowing for rapid fine-tuning of the function and stability of the MCL-1 protein in response to environmental and stress signals, consistent with the short half-life of the MCL-1 protein [13].

The two short isoforms, MCL-1S and MCL-1ES, display proapoptotic activity [14]. MCL-1S has only a BH3 domain, which represents a new pro-apoptotic BH3-only protein and is primarily located in the cytoplasm. Dimerization of MCL-1S and MCL-1L can antagonize the antiapoptotic effect of MCL-1L [15]. The splice variant MCL-1ES retains the BH3, BH1, and BH2 domains. It can induce mitochondrial apoptosis independently of BAK and BAX. MCL-1L can facilitate the proper localization of MCL-1ES oligomers on the mitochondrial outer membrane, and MCL-1ES neutralize the antiapoptotic activity of MCL-1L. These findings indicate that MCL-1ES may be a selective and effective target of MCL-1L in diseases that involve abnormal MCL-1L expression [16, 17]

### Regulation of MCL-1 expression

MCL-1 is subject to multiple modulations at the transcriptional, translational, and posttranslational levels. Many studies have suggested that multiple cytokines and signaling pathways are involved in the regulation of MCL-1. VEGF and interleukin-6 (IL-6) can regulate the expression of MCL-1 via autocrine signaling loops [18]. Activation of the ERK survival pathway prevents MCL-1 degradation and enhances its stability [19]. Activation of the Notch-1 signaling pathway induces the production of IL-6, further promoting the expression of MCL-1 [20]. MCL-1 protein levels are also regulated by cytokines, including IL-15 and IL-22, through the STAT3/MCL-1 [21] pathway or JAK/STAT and PI3K pathways [22].

Broad networks of miRNAs can regulate the expression of MCL-1. MiR-596 negatively regulates the MAPK/ERK signaling pathway by targeting MEK1 and regulates the apoptosis pathway by targeting MCL-1 and BCL-2 [23]. Upregulation of miR-15a/miR-16–1 leads to downregulation of the target genes BCL-2, MCL-1 and cyclin-D1, which directly leads to the death of leukemia cells [24, 25]. Other miRNAs, such as miR-26a [26], miR-15a, miR-101 and miR-197, can downregulate the expression of MCL-1 in vivo and inhibit cancer cell growth or apoptosis [27].

Stability of the MCL-1 protein can be controlled by a variety of E3 ubiquitin ligases, including MULE, SCFFbw7, APC/CCdc20 and SCFB-TrCP. These ubiquitin ligases effectively polyubiquitinate MCL-1 for degradation, while the deubiquitinases USP9X [28] and USP13 [29] stabilize expression of the MCL-1 protein. The PEST domain of MCL-1 contains many phosphorylation sites, such as Thr-92, Thr-163, Ser-64, Ser-155 and Ser-159. Phosphorylation of MCL-1 residues in the PEST domain by protein kinases, such as CDK1/2, GSK-3, JNK, and ERK, also affects the stability of MCL-1 [13, 30, 31].

### MCL-1 as a target for cancer therapy

**Fig. 3:**
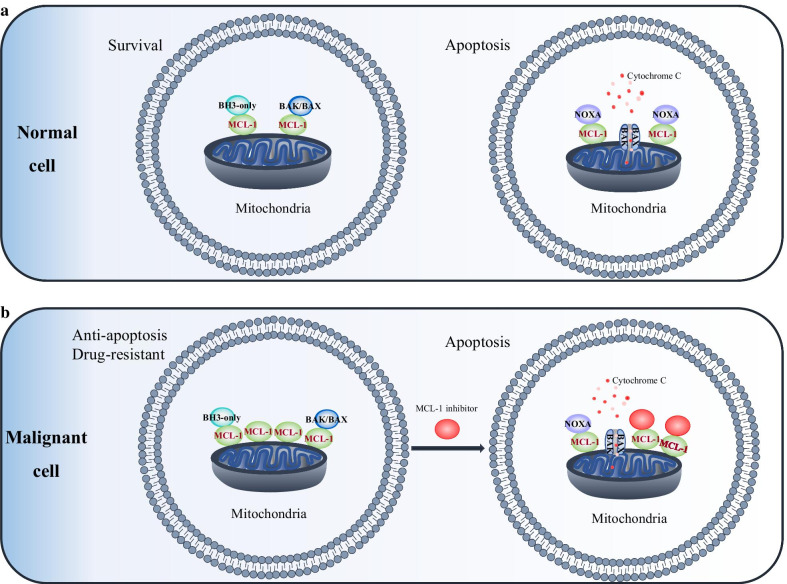
MCL-1 as a target for cancer therapy. **a** In normal cells, MCL-1 sequesters BH3-only proteins or neutralizes the effector proteins BAX and BAK, preventing cell death and maintaining cell survival. Various cell stress factors increase the expression of the special “activator” NOXA, subsequently replacing or preventing MCL-1 binding to BAX and BAK. BAX and BAK homo-oligomerize and form pores spanning the outer mitochondrial membrane to allow cytochrome C to be released into the cytoplasm, which triggers the activation of the caspase cascade and ultimately leads to cell apoptosis. **b** In malignant cells, overexpression of MCL-1 allows cancer cells to evade apoptosis by sequestering pro-apoptotic proteins. MCL-1 inhibitors selectively bind to MCL-1, freeing pro-apoptotic proteins, BAX/BAK, which initiates apoptosis

MCL-1 is a key survival factor for many cell types, allowing it to strictly regulate cell fate. In normal cells, MCL-1 sequesters the BH3-only activators BIM, BID and PUMA or neutralizes the effector proteins BAX and BAK, thereby antagonizing apoptosis [32] (Fig. 3a). When cells experience irreparable damage, they initiate an apoptotic program that increases the expression of pro-apoptotic BH3-only proteins, such as BIM, PUMA and NOXA [33, 34]. Then, BAX and BAK homopolymerize to form pores in the outer mitochondrial membrane, leading to infiltration of cytochrome c and other apoptotic proteins into the cytoplasm, promoting the formation of apoptotic microsomes, activation of caspases, cell lysis, and death [35, 36].

In recent years, many studies have shown that MCL-1 is essential for the survival and development of cancer cells. High levels of MCL-1 have been reported in hematological malignancies and a wide range of solid tumors [37, 38]. Overexpression of MCL-1 in cancer cells disrupts the balance between antiapoptotic and proapoptotic proteins, which prevents cancer cells from undergoing apoptosis, resulting in malignant proliferation [39] (Fig. 3b). Cancer has the capacity to develop multi-drug resistance against various therapies of different molecular pathways [40]. The mechanisms of resistance can be divided into intrinsic and acquired resistance. To escape apoptosis, cancer cells often express high levels of anti-apoptotic proteins and are "attracted" to them for their survival. Inhibition of one member of the BCL-2 anti-apoptotic family may cause dysregulation of the expression of other members. Increased expression of MCL-1 is a common response to long-term treatment with selective inhibitors of BCL-2/BCL-XL [41].Therefore, available evidences indicate that MCL-1 is an attractive target for cancer treatment.

### Strategies for the discovery of MCL-1 inhibitors

Many approaches have led to the development of MCL-1 inhibitors. Some drugs were not designed to specifically target MCL-1, but downregulate the expression of MCL-1 in an indirect way. For examples, cyclin-dependent kinase (CDK) inhibitors lead to decreased transcription of MCL-1, mTOR inhibitors block translation of MCL-1, and deubiquitinase inhibitors induce MCL-1 degradation through the proteasome system [42–44]. Here we mainly report strategies for the direct and selective inhibitors against MCL-1.

The realization that BH3-only proteins function as natural inhibitors of antiapoptotic BCL-2 proteins led to the development of BH3 mimetics. These BH3 mimetics bind competitively to the hydrophobic BH3-binding groove of antiapoptotic proteins, resulting in dissociation of proapoptotic BH3-only proteins or BAX/BAK and subsequent activation of apoptosis. Progress related to BH3 mimetics targeting BCL-2 and BCL-xL has been made [45], such as ABT-737, which binds BCL-2, BCL-xL, and BCL-W [46]; ABT-263 (navitoclax), which binds BCL-2, BCL-xL [47], and venetoclax (ABT-199), which selectively binds BCL-2 [48] and has been approved by the FDA for chronic lymphocytic leukemia (CLL) treatment.

However, most BCL-2 family inhibitors cannot bind to highly differentiated MCL-1 molecules. Upregulation of MCL-1 has been shown by several studies to be a major limiting factor in the development of ABT-737 and ABT-263 resistance in tumor treatment. The creation of high-affinity inhibitors that directly target MCL-1 remains a challenge. Furthermore, MCL-1 has a BH3-binding groove that lacks plasticity. The binding pockets on MCL-1 are shallow and relatively inflexible compared to those of BCL-2 and BCL-xL. Increasing structural studies demonstrate that MCL-1 interact with the BH3-only proteins through a canonical mechanism similar to that of BCL-2 and BCL-xL, in which four hydrophobic residues (h1, h2, h3, and h4) of the BH3 helix of BH3-only proteins respectively interact with four pockets (P1–P4) in the hydrophobic binding groove of MCL-1 (Fig. 2c, d), and a common salt bridge is formed between an Asp of BH3-only proteins and Arg263 of MCL-1[12, 49]. The P1-P4 pockets and Arg263 are considered as hot-spots of MCL-1 to design BH3 mimetics [50].

Although there are many challenges in the process of new drug research and development, the most fundamental is the discovery and optimization of lead compounds [51, 52]. High-throughput screening (HTS) and virtual screening are widely used for the discovery of lead compounds [53, 54]. Molecular modeling can predict the binding mode of small molecules to target proteins, which may facilitate the optimization of small molecule drugs and the development of MCL-1 inhibitors with higher safety and efficacy [53, 54]. In recent years, more strategies have been applied to the screening and optimization of MCL-1 inhibitors, such as NMR-based fragment screening, computational modeling and fragment-based design.

In the following sections, we summarize the current discoveries of direct and selective inhibitors of MCL-1, including the current status and future applications of these small molecule inhibitors, as well as their use alone and in combination to treat a variety of cancers.

### Direct and selective inhibitors targeting MCL-1

#### MCL-1 inhibitors in clinical trials

In recent years, considerable progresses has been made with potent and highly selective MCL-1 inhibitors. Until now, six compounds have entered phase 1 clinical trials and they have been shown to induce cancer cell apoptosis in preclinical trials (Table 1).

**Table 1:** MCL-1 targeted anti-tumor drugs in clinical trials

Agents	Frist report time (drug discovery method)	Identifier/phase	Population	Regimen
*Monotherapy*				
S64315 (MIK665)	2016 (NMR-based fragment screen)	NCT02979366 Phase 1	AML, MDS	Once or twice a week (21/28-day cycle), the starting dose is 50 mg (intravenous)
		NCT02992483 Phase 1	MM, DLBCL	Dose finding (intravenous)
AZD5991	2018 (NMR-based fragment screen and Structure-based design)	NCT03218683 Phase 1	AML, CLL, MDS, MM	Intravenously for 9 cycles (21-day cycle)
AMG-176	2016 (High-throughput screening)	NCT02675452 (Suspended)Phase 1	MM, AML	Dose finding(intravenous)
AMG-397	2018 (-)	NCT03465540 (Suspended)Phase 1	MM, NHL, AML, DLBCL	Once a day for 2 consecutive days followed by 5 days break at a weekly interval (28-day cycle) (oral)
ABBV-467	NA	NCT04178902 Phase 1 2019	MM and Cancer	Dose finding (intravenous)
PRT1419	NA	NCT04543305 Phase 1 2020	MM, NHL, AML, MDS	Dose finding (oral)
*Combination therapy*				
S64315 + Venetoclaxv		NCT03672695 Phase 1	AML	S64315 once a week (intravenous) Venetoclax once a day (oral) (21-day cycle)
AZD5991 + Venetoclaxv		NCT03218683 Phase 2	AML, MDS	Ascending oral doses of Venetoclax
AMG-176 + Venetoclaxv		NCT03797261 (Suspended)Phase 1	AML, NHL/DLBCL	Two-consecutive days per week (QD2)

*RS* Richter syndrome, *SLL* Small lymphocytic lymphoma

### S63845/S64315

**Fig. 4:**
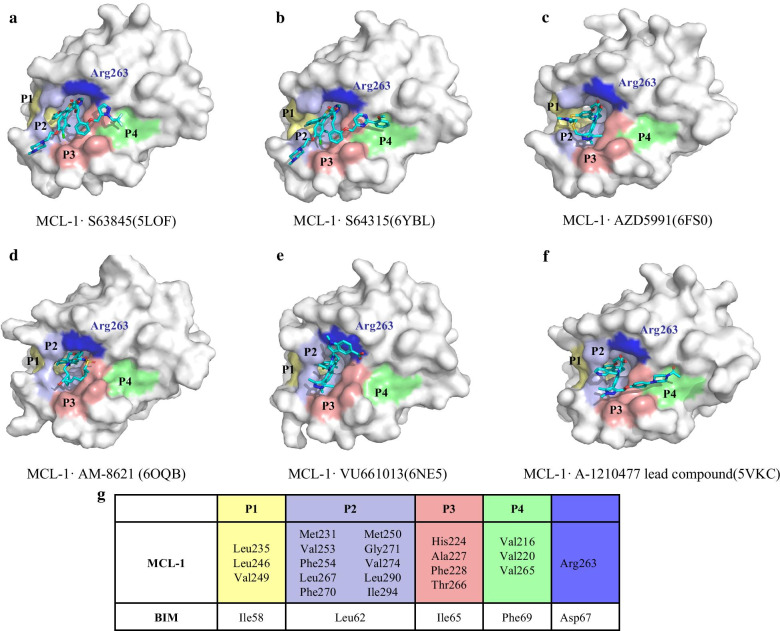
Co-crystal structures of MCL-1 in complex with its inhibitor. **a** MCL-1/S63845 complex (PDB: 5LOF), **b** MCL-1/S64315 complex (PDB: 6YBL), **c** MCL-1/AZD5991 complex (PDB: 6FS0), **D.** MCL-1/AM-8621 complex (PDB: 6OQB), **e** MCL-1/VU661013 complex (PDB: 6NE5), **f** MCL-1/A-1210477 lead compound complex (PDB: 5VKC). The four hydrophobic pockets (P1-4) and Arg263 are highlighted and labelled. **g** MCL-1 hot-spots residues for P1-P4 pockets and the four hydrophobic residues of BIM (h1, h2, h3, h4) based on the MCL-1/BIM structure (PDB: 2NL9)

S63845 is a potent and selective small molecule inhibitor of MCL-1 discovered by NMR-based fragment screening. It inhibits MCL-1 with a *K*_*i*_ < 1.2 nM and Kd of 0.19 nM and has no evident binding to BCL-2 or BCL-xL (*K*_*i*_ > 10,000 nM) [55]. The structure of S63845 in complex with MCL-1 (PDB: 5LOF) shows that S63845 forms a salt bridge with Arg263 via a carboxyl group, while its aromatic scaffold stretches deep into the P2 pocket and a terminal trifluoromethyl group extends into the small P4 pocket, with some P1 residues constituting part of the P2 pocket (Fig. 4a) [55].

S64315 (MIK665) belongs to the same series of compounds as S63845 [56]. The filling of the P2 pocket and the extension of the P4 pocket increased the activity of S64315 (PDB: 6YBL) compared to S63845 (Fig. 4b) [57]. S64315 is currently in a phase 1 clinical trial to evaluate the maximal tolerated dose and the recommended dose for expansion. A further study will evaluate the tolerability, safety, and antitumor activity of MIK665 for the treatment of refractory or recurrent lymphoma and multiple myeloma (MM) (NCT02992483) [58], acute myeloid leukemia (AML), and myelodysplastic syndrome (MDS) (NCT02979366) [59]. A dose-escalation study of S64315 in combination with venetoclax for AML is also underway (NCT03672695) [60].

### AZD5991

AZD5991 is a macrocyclic molecular inhibitor with high selectivity and affinity for MCL-1 (*K*_*i*_ = 200 pM, IC_50_ = 0.72 nM) [61]. AstraZeneca researchers designed and synthesized AZD5991 by analyzing indole-2-carboxylic acid derivatives previously reported by the Abbvie [62] and Fesik's laboratory [63]. The co-crystal structure (PDB: 6FS0) shows that AZD5991 binds largely in the P2 and P3 pockets (Fig. 4c) [61]. Its naphthalene ring penetrates deep into the P2 pocket, and the indole core interacts mainly with P3 residues. The methyl group distal to the indole core makes contact with P1 pocket. Moreover, the carboxylic acid orthogonal to the indole ring makes strong salt-bridge interactions with Arg263 [61]. Currently, clinical trials of single-drug intravenous injection of AZD5991 and in combination with venetoclax are ongoing in patients with recurrent or refractory hematologic malignancies (NCT03218683) [64].

### AMG-176

AMGEN screened compound 1 from 248,090 compounds using HTS method, and subsequently optimized to obtain AMG-176 using structure-based design. AMG-176 is a potent MCL-1 inhibitor (*K*_*i*_ = 0.06 nM) and shows little affinity for BCL-2 (*K*_*i*_ = 0.95 µM) and BCL-xL (*K*_*i*_ = 0.7 µM) [65]. The structure of MCL-1 complexed with AM-8621 (PDB: 6OQB), a ligand structurally similar to AMG-176, shows that the inhibitor primarily makes contact with P2 and P3 pockets, whereas no hydrogen bond or salt bridge is observed between the carbonyl group and Arg263 (Fig. 4d) [65]. Recent reports [66, 67] show that additional hydrogen bond between the carbonyl group of AMG-176 and Arg263 of MCL-1 would allow for tighter interactions in the P3 pocket. AMG-176 was first subjected to relapsed or refractory MM or AML in a human clinical trial to obtain an intravenous dose of AMG-176 that is safe and tolerable for the patient (NCT02675452) [68]. Another clinical trial of AMG-176 combined with venetoclax is also currently underway [69].

### AMG-397

Based on the chemical structure of AMG-176, a structure-guided approach in combination with ligand-based design was used to obtain AMG-397 with higher affinity and antitumor activity [66, 67]. AMG-397 is the first oral MCL-1 inhibitor to reached the clinical trial stage. It inhibits MCL-1 with a *K*_*i*_ of 15 pM and interferes with the interaction of MCL-1 with BIM in cells [66]. A clinical trial is evaluating AMG-397 in patients with MM, AML, and non-Hodgkin's lymphoma (NHL). AMG-397 was administered orally two days a week, followed by a five-day break. However, due to safety signals of cardiotoxicity, a phase 1 dose escalation clinical trial is being put on hold (NCT 03465540) [70]. Based on this safety issue, the first phase of the AMG-176 trial has also been voluntarily suspended.

### ABBV-467

ABBV-467 is a selective MCL-1 inhibitor. Currently, there are few reports on ABBV-467, but it is being studied in a phase 1 clinical study (NCT04178902) [71]. This study will evaluate the safety and tolerability of ABBV-467 in adult participants with relapsed/refractory MM.

### PRT1419

PRT1419 is a potent, selective oral inhibitor of MCL-1 developed by Prelude Therapeutics. Prelude Therapeutics claims that PRT1419 specifically binds MCL-1 in preclinical models of hematologic tumors and shows efficacy. Phase I dose expansion study of PRT1419 in relapsed/refractory hematologic malignancies currently underway (NCT04543305) [72].

#### Preclinical MCL-1 inhibitors

Currently, although a small number of MCL-1 inhibitors have entered clinical trials, no drugs have been approved for clinical use. Given the prominence of MCL-1 inhibitors in cancer therapy, researchers have utilized various approaches to identify a cohort of MCL-1 inhibitors (Table 2), which are expected to enter clinical trials.

**Table 2:** MCL-1 targeted anti-tumor drugs in the preclinical stage

Compound		Efficacy towards malignant cells	In vitro potency	drug discovery method	References
MIM1		MCL-1 dependent leukemia cells	IC_50_: < 4.2 μM	HTS (2013)	[81]
UMI-59/77		BxPC-3 xenograft model	K_i_: 490 nM	HTS (2014)	[84]
Complex 39		NCI-H460 cell line and xenograft model	IC_50_: 12–18 μM		[102]
Complex 14		NCI-H460 xenograft model	Ki: 1.4 nM		[101]
Pyridoclax/Compound12		Ovarian cancer cell line	–	(2014/2018)	[77, 78]
ML311/EU-5346		Active MCL-1 cell line	IC_50_: 0.31 μM	HTS (2013)	[82, 83]
A-1210477		MCL-1-dependent cell lines	*K*_*i*_: 4–5 nM	HTS (2015)	[62]
BIM SAHB_A_	NA	DLBCL cell lines	EC_50_: 2–18 μM		[79]
MS1	PREIWMTQGLRRLGDEINAYYAR	MCL-1 dependent cell lines	*K*_*d*_:1.9 ± 1.0 nM		[80]
VU661013		AML, MM, triple negative breast cancer cell lines	*K*_*i*_: 97 ± 30 pM	Structure-based design (2018)	[90, 124]
Wang.Compound12		NCI-H345 cell line	IC_50_: 2.2 μM	Fragment-based approach (2016)	[107]
Compound8		A2780, MCF-7, SMMC-7721 and DLD1 cell lines	IC_50_: 38–47 μM	Virtual screening (2017)	[108]
Compound24		Lymphoma cell lines	*K*_*i*_: 100 nM	HTS and virtual screening (2020)	[109]
Compound M08		Hematological and solid cancer cell lines	K_i_: 0.53 ± 0.07 μM	Structure-based virtual screening (2020)	[110]
Compound5		L-363, LP-1, NCI-H929 and MOLP-8	IC_50_: 3.4 nM		[92]
C3		HeLa, K562, NCI-H23 cell line	IC_50_: 0.78 ± 0.12 μM	(2019)	[105]
dMCL1-2		MM cell line OPM2	*K*_*d*_: 30 nM	(2019)	[104]
MI-223		H1299 cell	*K*_*d*_: 193 ± 4.3 nM	Virtual screening (2018)	[91]
APG-3526	NA	MM cell line	IC_50_: 7 nM	(2020)	[106]

*EC*_*50*_ Half-maximal concentration of drug, *IC*_*50*_ Concentration inhibitory to 50% of cells, *K*_*i*_ Inhibition constant, *K*_*d*_ Dissociation constant

### NOXA-like compound

NOXA is a naturally occurring, highly selective MCL-1 inhibitor [73]. NOXA can competitively bind MCL-1 and release BAX and BAK, which originally bind to MCL-1. NOXA has been reported to enhance MOMP and apoptosis in a variety of cancers, such as glioblastoma [74], cholangiocarcinoma [75], and chronic myeloid leukemia [76]. Hedir and colleagues identified a compound12 with polar substituents that can bind to the inside of the MCL-1 pocket in a NOXA-like manner, thereby releasing BIM and BAK and inducing cell apoptosis [77, 78].

### BIM-BH3 peptide

The BH3 domain of BIM can bind to the BH3 hydrophobic groove of BCL-2 antiapoptotic proteins and directly activate the apoptotic effector proteins BAK and BAX. BIM-SAHB_A_ is a hydrocarbon-stapled peptide based on the BH3 structural domain of BIM, and its primary intracellular target is MCL-1 [79]. MCL-1 knockout mouse embryonic fibroblasts are resistant to apoptosis induced by BIM-SAHB_A_ at the mitochondrial level. Screening of the yeast surface display library of the BIM-BH3 identified the MCL-1-specific peptide MS1 [80]. MS1 induces apoptosis in a variety of MCL-1-dependent tumor cells with higher sensitivity than NoxaA [80].

### MIM1, ML311/EU-5346 and UMI-77

MIM1 was obtained from 71,296 compounds by HTS with the help of fluorescence polarization assay [81]. ML311/EU-5346 was confirmed from 315,100 compounds by HTS, which interfered with the MCL-1/BIM interaction and induced cell death in MCL-1-dependent cell lines (EC_50_ = 0.3 μM) [82, 83]. Fardokht et al. identified and verified UMI-59 from 53,000 compounds by HTS method [84]. UMI-77 is the product of further chemical modification of UMI-59 and has a higher affinity for MCL-1, with *K*_*i*_ = 0.49 µM [84]. These inhibitors were predicted to bind at the P2 and P3 pockets, while forms a hydrogen bond with Arg263 [81, 84].

### A-1210477

Abbvie identified indole acid derivative that selectively bind MCL-1 by HTS method combined with fluorescence polarization assay, which were later optimized to yield A-1210477 [62]. A-1210477 has a potent affinity for MCL-1 (*K*_*i*_ = 4–5 nM). The structure of a close analog complexed with MCL-1 (PDB: 5VKC) indicates that it forms a typical hydrogen bond with Arg263, and binds to P2, P3 and P4 pockets with the naphthyl ring stretching deep into P2 pocket (Fig. 4e) [62]. A-1210477 disrupts the interaction between intracellular BIM and MCL-1 and promotes apoptosis in a mitochondria-dependent manner [62, 85].

### VU661013

VU661013 is established after a series of structural optimizations, which has an effective and selective activity to MCL-1 (*K*_*i*_ = 97 ± 30 pM). It interferes with the binding stability of BIM and MCL-1 [63, 86–89]. The co-crystal structure MCL-1/VU661013 complex (PDB: 6NE5) reveals that the dimethyl chlorophenyl ether of VU661013 inserts deep into P2 pocket, with the methyl group of the trimethyl pyrazole pointing towards P3 pocket (Fig. 4f) [90]. Notably, its indole headpiece makes a cation-*π* interaction with Arg263, and positions the carboxylic acid to form a favorable hydrogen bond with Asn260 [90].

### MI-223

Besides anti-apoptotic functions, MCL-1 is also involved in the regulation of non-apoptotic functions, including mitochondrial homeostasis, cell cycle regulation, DNA damage repair and autophagy [43]. MCL-1 interacts with the dimeric complex of Ku proteins through its BH1 and BH3 domains to inhibit Ku protein-mediated non-homologous end-joining and promote homologous recombination-mediated DNA repair [91]. Based on this mechanism, a new small molecule compound MI-223 was discovered, which directly binds to the BH1 domain of MCL-1 (*K*_*i*_ = 193 ± 4.3 nM), disrupts MCL-1 and Ku protein interactions.

### Covalent inhibitory

Covalent inhibitors exert biological functions by interacting with target protein residues through covalent bonds. Therefore, they have the advantages of high selectivity, strong affinity, low effective drug concentration and low possibility of resistance. Akçay et al. [92] analyzed the crystal structure of MCL-1 and concluded that Lys234 was likely to be covalently modified. Subsequently, they used aryl boronic acid to covalently modify the noncatalytic lysine residues. In a panel of compounds, compound 5 had an IC_50_ of 3.4 nM, and compound 11 directly interfered with the MCL-1/BAK interaction in the cell. After mutation of the lysine 234 site, no covalent compounds were formed by mass spectrometry analysis, further verifying that Lys234 was covalently modified. Additionally, the efficacy of this reversible covalent inhibitor in vivo needs to be further refined.

### Natural products

Some drugs extracted from natural products are characterized by high biological activity and low toxicity [93]. Gapil et al. extracted 26 carboxamides from natural fislatifolic acid, one of which exhibited submicromolar affinity for MCL-1 and BCL-2, and showed moderate cytotoxicity in lung and breast cancer cell lines [94]. Meiogynin A1 inhibited BCL-xL and MCL-1, and had no toxic effects on normal cells [95]. The modification of side chains gives these compounds better affinity and antitumor activity [96]. Cryptosphaerolide inhibited the interaction between MCL-1 and BAK and showed strong inhibitory activity against colon cancer cell line HCT-116 with an IC_50_ of 4.5 μM [97]. Maritoclax selectively inhibits the proliferation of leukemic cells with high MCL-1 expression and significantly enhances the therapeutic effect of ABT-737 on various hematological malignancies [98, 99].

### Metal-based complexes

Copper is essential element that participate in the reactions of various enzymes in the body, so copper-based complexes are promising anti-cancer drugs [100]. Lu et al. designed and synthesized a series of copper(II) complexes of 9-substituted β-carboline [101]. Complex 14 was able to selectively inhibit MCL-1 and disrupt MCL-1/BAX and MCL-1/BAK complexes in tumor cells, inducing BAX/BAK-dependent apoptosis in tumor cells. Structural model indicates that complex 39 can interact with Arg263 of MCL-1 while the long alkyl chain inserted into the P2 pocket of MCL-1 [102]. These findings have laid the foundation for the development of metal-based MCL-1 inhibitors.

### MCL-1 PROTACs

Proteolysis-targeting chimeras (PROTACs) are a novel class of drug molecules designed to degrade proteins. They consist of three parts: an E3-binding ubiquitin ligase, a moiety that binds to the target protein, and a linker group that connects the two [103]. Recruitment of E3 ligase to the target protein can lead to proteasomal ubiquitination and subsequent degradation of the target protein. Structure–activity relationship (SAR) studies have yielded dMCL1-2 (Kd = 30 nM) [104] and C3 (IC_50_ = 0.78 ± 0.12 μM) [105]. Through the formation of a "target protein-PROTAC-E3 ligase" complex, the affinity between MCL-1 and the E3 ligase CRBN is effectively enhanced, which selectively degrades MCL-1 to obtain stronger tumor cell killing activity.

### Other compounds

Ascentage Pharma has recently identified two lead compounds for MCL-1 inhibitors, APG-3526 and AS00491, using a protein–protein interaction platform [106]. In vitro and in vivo studies demonstrates that APG-3526 (IC_50_ = 7 nM) and AS00491 have high affinity for MCL-1 and anti-tumor proliferative capacity [106]. There are also many other preclinical MCL-1 inhibitors [102, 107–110] (Table 2). These compounds aim to promote MCL-1-dependent cancer cell apoptosis and exhibit differential activity.

### Targeting MCL-1 in different cancers

Evasion of apoptosis through dysregulation of BCL-2 family is a significant hallmark of tumorigenesis and drug resistance. Different types of tumors determine the sensitivity to MCL-1 inhibitors by the expression of MCL-1. MCL-1 inhibitors can restart apoptosis when in combination with other therapies, allowing various cancer types to benefit from it. Here, we present the pathological and treatment role of MCL-1 in different kinds of cancers.

### Multiple myeloma

Gain or amplification of chromosome 1q21 encoding MCL-1 and IL-6 receptor in patients with MM is associated with significantly shorter progression-free survival [111]. MCL-1 protein expression increased in newly diagnosed MM patients, with higher levels in relapsed patients [112]. Most MM cell lines are dependent on MCL-1 for survival, and targeting MCL-1 induces apoptosis in approximately 70% of myeloma cell lines [113]. Cell-dependent analysis of 33 human MM cell lines showed a significant increase in MCL-1 dependence from 33% at diagnosis to 69% at relapse, suggesting that MCL-1 cell dependence favors relapse [114].

Targeting MCL-1 represents a novel and effective strategy for the treatment of MM. S63845 showed high efficacy and sensitivity in a panel of MM cell lines with IC_50_ < 0.1 μM, and exhibited dose-dependent antitumor activity in MM xenograft mice. Moreover, S63845 may be effective in cases with refractory/relapsed MM or drug resistance, as its efficacy is not limited to patients carrying chromosomal translocations or mutations. 100 days after treatment of the AMO1 (MM cell line) model with 25 mg/kg dose of S63845, seven mice (eight in total) showed complete tumor regression [55].

AZD5991 directly binds to MCL-1 in MOLP-8 (MM cell line) cells and releases BAK from the MCL-1/BAK complex within 15 min at a concentration of 10 nM [61]. After a single intravenous injection of 10 mg/kg, 30 mg/kg, and 100 mg/kg AZD5991, xenograft models of MM mice showed 52%, 93%, and almost complete 100% tumor regression, respectively [61].

In MM xenograft models, oral administration of AMG-176 resulted in BAK activation, cleaved caspase 3, and cleaved poly-ADP ribose polymerase (PARP) within 2 h [65]. Also in MM mice, oral administration of AMG-176 at a daily dose of 60 mg/kg or once-weekly dose of 100 mg/kg achieved 100% tumor growth inhibition and 97% regression, respectively. The combination of AZD5991 or AMG-176 with venetoclax or proteasome inhibitors achieves a more potent antitumor killing effect than either single agent in MM [61, 65]. In MM xenografts models, AMG-397 administered orally once or twice weekly at 25 or 50 mg/kg exhibited significant tumor regression [66]. Of these, nine mice (ten in total) in the dose of 50 mg/kg had complete tumor regression at the end of the study [66].

### Leukemia

AML usually exhibits heterogeneous expression of antiapoptotic proteins, especially MCL-1 [115]. Detection of BCL-2, BCL-xL and MCL-1 expression in hematopoietic progenitor cells and leukemic cells from AML patients revealed that MCL-1 transcripts were expressed at high levels in all samples tested [116]. MCL-1 is a key survival molecule required to promote the survival of B progenitor cell populations during BCR-ABL transformation and the continued survival of BCR-ABL B-acute lymphoblastic leukemia cells [117]. MCL-1 overexpression has been identified in chemotherapy-relapsed AML and is a major factor in resistance to the dual BCL-2/BCL-XL inhibitor ABT-737 in AML cell lines [118]. MCL-1 and BCL-xL can make cells resistant to the BCL-2 inhibitor ABT-199 [119].

MCL-1 inhibition can be a rational therapeutic approach against AML. All eight AML cell lines were sensitive to S63845 treatment with IC_50_ values of 4–233 nM [55]. AML xenograft model mice treated with 25 mg/kg dose of S63845 for 80 days resulted in complete remission in 6 out of 8 mice [55]. Compared to other BH3 mimics, such as ABT-199/A1331852 (EC_50_ < 3 μM), S63845 is more effective in killing primary AML cells and their derived cell lines (EC_50_ < 150 nM) and has the least toxicity to CD34 + progenitor cells [120].

AZD5991 induced apoptosis only in MCL-1-dependent cells. In AML mouse models, once weekly intravenous injection of 60 mg/kg AZD5991 reduced leukemic cells in both peripheral blood and bone marrow, while daily oral administration of 100 mg/kg venetoclax only reduced leukemic cells in peripheral blood [61]. For AML cells resistant to either single agent, AZD5991 combination with Venetoclax can overcame resistance without significant changes in body weight [61]. The synergistic effect of AZD5991 with penatinib or venetoclax results in a new clinical choice for patients with T315I (+) Ph + leukemia [121].

In the MOLM-13 luciferase labeled AML model, oral AMG 176 at a dose of 60 mg/kg twice weekly resulted in 69% tumor regression [65]. Treatment of MOLM-13 mice with AMG-176 (30 mg/kg) twice weekly or with venetoclax (50 mg/kg) daily both significantly reduced tumor load by 55% and 23%, respectively. Synergistic use demonstrated 100% complete tumor suppression compared to monotherapy. Similar results were obtained in samples of patients with primary AML. In 9 of 13 samples, the combination of equimolar concentrations of AM-8621 and venetoclax significantly inhibited tumors compared to either drug alone [65]. Compared to DMSO, AMG-176 caused a negligible number of normal hematopoietic cell deaths [122].

AMG-397 showed a strong sensitivity to AML cell lines. In the MOLM-13 orthotopic model of AML, twice-weekly dosing at 30 mg/kg achieved 99% tumor growth inhibition. The combination of oral AMG-397 at 10 mg/kg twice weekly and venetoclax at 50 mg/kg daily achieved 45% regression [66].

A-1210477 overcomes the resistance of AML mouse models and cell lines to the BCL-2/BCL-XL inhibitor agent (ABT-737) [123]. VU661013-resistant AML cells were significantly more sensitive to venetoclax than their initial response and that cells resistant to venetoclax were more sensitive to MCL-1 inhibitors [124]. AML mice treated with VU661013 (75 mg/kg daily) for 3 weeks died of AML 42 days later. But with venetoclax effectively induced apoptosis [124].

### Non-Hodgkin lymphoma

MCL-1 is highly expressed in malignant B cells and aggressive B-NHL [125]. MCL-1 transgenic mice develop B-cell lymphoma at high frequency [126]. MCL-1 is important for the survival of B lymphocyte progenitor cells in MYC-driven lymphomagenesis. Overexpression of MCL-1 greatly accelerates the development of lymphoma driven by the oncogene c-MYC. Although the loss of one MCL-1 allele does not significantly impair the survival of normal B lymphocyte-like cells, it almost completely abrogates the development of MYC-driven lymphomas [127]. MCL-1 knockdown triggers spontaneous apoptosis in several mantle cell lymphoma cell lines [128]. In two different mantle cell lymphoma cell lines, one normal cell line (JeKo-1) and one invasive cell line (MAVER-1), silencing MCL-1 induced a dose-dependent increase in the proportion of apoptotic cells [129].

In a human MCL-1 murine lymphoma transplant model, a single dose of S63845 at 12.5 mg/kg or cyclophosphamide at 50 mg/kg in combination with S63845 at 7.5 mg/kg inhibited tumor growth by 60% and almost 100%, respectively [130]. S63845 combined with BCL-2/BCL-xL inhibitors exhibited improved antitumor activity in B-cell acute lymphoblastic leukemia [131].

BIM-SAHB_A_ can induce the apoptosis of diffuse large B cell lymphoma (DLBCL) (EC_50_ = 2–18 μM), regardless of the expression of antiapoptotic proteins, and it is most effective among DLBCL with resistance to ABT-737 and ABT-199 [79]. In BCL-2^High^ NHL cell lines, both A-1210477 and CDK inhibitors downregulated MCL-1 expression and induced apoptosis in synergy with Venetoclax. In most BCL-2^Low^ NHL cell lines, A-1210477 also exerted synergistic effects with navitoclax [132].

### Lung cancer

Munkhbaatar et al. validated the high-frequency of MCL-1 in lung adenocarcinoma in multiple open databases [133]. MCL-1 overexpression is associated with poor survival in non-small cell lung cancer [134]. The PEST domain of MCL-1 interacts with AKT on the PH domain to activate AKT, which together promote lung cancer progression [135]. Lung cancer models with stable expression of BCL-xL and MCL-1 treated with radiotherapy induce negligible numbers of apoptotic cells [136].

S63845 induced cytotoxicity in lung adenocarcinoma cell lines is positively correlated with MCL-1 protein expression, which delays tumor progression and reduces tumor size in mouse [133]. MCL-1 inhibition can be used in combination with other therapeutic strategies to lower the apoptosis threshold [133]. MI-223 in combination with olaparib significantly inhibited lung tumor growth without hematological or histopathological toxicity [91]. Metal-based complex 14 and 39 significantly inhibited tumor growth in the NCI-H460 xenograft model [101].

### Breast cancer

Amplification of MCL-1 is more frequent in clinical breast cancer datasets than BCL-2 and BCL-xL, and is associated with poor prognosis [137]. In vitro and in vivo experiments demonstrated the dependence of triple-negative breast cancers (TNBC) on MCL-1 [137]. HER2-positive breast cancer has significantly lower NOXA levels and mediates resistance to HER2 inhibitors through upregulation of MCL-1 [138].

In breast cancer with high expression of MCL-1, S63845 displayed synergistic activity with docetaxel in TNBC and with trastuzumab or lapatinib in HER2-amplified breast cancer [139]. Combined inhibition of BCL-2 and BCL-xL with ABT-263 had limited efficacy on breast cancer owing to high expression of MCL-1, while A-1210477 or VU661013 in combination ABT-263 has a synergistic effect [140, 141]. EU-5346 in combination with paclitaxel induced synergistic activity in both paclitaxel-sensitive and paclitaxel-resistant TNBC cells [142].

### Colorectal cancer

MCL-1 expression is significantly increased in colorectal cancer and is associated with tumor stage, lymph node metastasis, and poor prognosis [143]. MCL-1 inhibitors synergize with standard therapies to exert antitumor activity in colorectal cancer. Decreased degradation of MCL-1 is involved in the E3 ubiquitin ligase FBW7 mutation-induced resistance to regorafenib in colorectal cancer patients [144]. The MCL-1 inhibitor S63845, AZD5991, AMG-176 restore sensitivity to regorafenib in FBW7 mutant colorectal cancer cells by restoring the apoptotic response [145]. In BRAF^V600E^-mutant colorectal cancer, mutant BRAF upregulates MCL-1 to confer apoptosis resistance [146]. MCL-1 inhibitor A-1210477 in combination with cobimetinib reverses colorectal cancer drug resistance and enhances cobimetinib-induced apoptosis [146].

### Melanoma

Increased MCL-1 expression through oncogenic activation of BRAF was observed in cutaneous metastatic melanoma [147]. MCL-1 depletion significantly induced apoptosis in melanoma cells and resensitized mutant BRAF melanoma cells to anoikis compared with depletion of BCL-2 or BCL-xL [148]. Combined inhibition of MCL-1 and BCL-xL by S63845/S64315 plus Navitoclax [149] or the combination of MCL-1 and BCL-2 by S63845/S64315 plus ABT-199 [150] synergistically induces extensive death in advanced/refractory melanoma cell lines both in vitro and in vivo. MIM1 promotes mitochondrial membrane rupture, glutathione depletion and cell cycle arrest, inducing melanoma cell death [151, 152].

### Hepatocellular carcinoma

MCL-1 is a survival factor for hepatocellular carcinoma (HCC) [153]. MCL-1 expression was enhanced in HCC cell lines as well as in human HCC tissues. High expression of MCL-1 inhibits JQ1-triggered apoptosis in HCC cells [154]. MCL-1 knockdown or specific inhibitors of S63845 or A-1210477 significantly inhibited hepatocellular carcinoma spheroid cell formation and triggered apoptotic signals [153]. Targeting MCL-1 directly promoted apoptosis of hepatoma cells without affecting the growth of normal hepatocytes [155]. A combination of inhibitory CDK can overcome the resistance of hepatocellular carcinoma cells to sorafenib, and CDK-mediated inhibition of MCL-1 plays a key role in mediating this process [156].

### Other solid tumors

The dependence of solid tumors on MCL-1 may be responsible for drug resistance. MCL-1 small molecule inhibitors, such as MIM1, UMI-77, and A-1210477, in combination with other standard therapies may be an effective strategy to restore the sensitivity of resistant cells, including head and neck squamous cell carcinoma [157], glioblastoma [158], cervical cancer [159], pancreatic cancer [84], ovarian cancer [160], and esophageal squamous cell carcinoma [161]. Thus, MCL-1 is a potential therapeutic target for restoring cell apoptosis in multidrug-resistant cancers. MCL-1 inhibitors in combination with existing radiotherapy/chemotherapy can overcome resistant/relapsed tumors, increasing the disease-free survival of cancer patients.

## Conclusions

MCL-1 plays important roles in cancer development, and is associated with drug resistance of a variety of cancers. In recent years, significant progress has been made with MCL-1 inhibitors, and some drugs have entered clinical trials. In this review, we present a comprehensive summary of inhibitors that selectively target MCL-1, including small molecule inhibitors, peptide inhibitors, covalent inhibitors, natural products, metal-based complexes, and MCL-1 PROTACs. We analyze these inhibitors in terms of screening methods, chemical structures, binding modes and co-crystal structures. In addition, we discuss the use of MCL-1 selective inhibitors in different hematologic malignancies and solid tumors. In 2016, the FDA approved the BCL-2 selective inhibitor venetoclax for the treatment of chronic lymphocytic leukemia. As the clinical development of MCL-1 selective inhibitors progresses, MCL-1 selective inhibitors may become a new class of anti-cancer drugs that will bring clinical benefits to patients with a variety of hematologic malignancies and solid tumors.

## Data Availability

Not applicable.

## Abbreviations

AML: Acute myeloid leukemia
BH: BCL-2 homologous domains
CDK: Cyclin-dependent kinases
CLL: Chronic lymphocytic leukemia
DLBCL: Diffuse large B-cell lymphoma
HCC: Hepatocellular carcinoma
HTS: High-throughput screening
MCL-1: Myeloid leukemia 1
MDS: Myelodysplastic Syndrome
MM: Multiple Myeloma
MOMP: Mitochondrial outer membrane permeabilization
NHL: Non-Hodgkin lymphoma
PARP: Poly-ADP ribose polymerase
PROTACs: Proteolysis-targeting chimeras
RS: Richter syndrome
SLL: Small lymphocytic lymphoma
TNBC: Triple-negative breast cancers
